# Kangfuxiaoyanshuan alleviates uterine inflammation and adhesion via inhibiting NF-κB p65 and TGF-β/MMP-2 signaling pathway in pelvic inflammatory disease rats

**DOI:** 10.3389/fphar.2022.894149

**Published:** 2022-07-18

**Authors:** Linyuan Fan, Zhaohui Liu, Zhan Zhang, Ting Li, Xiaonan Zong, Huihui Bai

**Affiliations:** ^1^ Department of Gynecology, Beijing Obstetrics and Gynecology Hospital, Capital Medical University. Beijing Maternal and Child Health Care Hospital, Beijing, China; ^2^ Department of Microecological Laboratory, Beijing Obstetrics and Gynecology Hospital, Capital Medical University. Beijing Maternal and Child Health Care Hospital, Beijing, China

**Keywords:** pelvic inflammatory disease (PID), kangfuxiaoyanshuan, anti-inflammation, TGF-β, MMP-2, NF-κB

## Abstract

**Background and aims:** Pelvic inflammatory disease (PID) is infection-induced inflammation of the female upper reproductive tract that results in high fever, ectopic pregnancy, infertility, and varying degrees of chronic pelvic pain. Recent clinical studies have shown that Kangfuxiaoyanshuan (KFXYS), a Traditional Chinese Medicine (TCM) formulation, may short the course of the disease and reduce the occurrence of PID sequelae, but its pharmacological action and potential mechanism have not been fully elucidated. Here, we aimed to investigate the therapeutic effects and mechanism of KFXYS in rats with PID.

**Materials and Methods:** A PID rat model was constructed through endometrial mechanical injury and pathogen infection. The rectal temperature was measured during the 14-days course of treatment, and the white blood cell (WBC) count in the blood and the levels of cytokines (IFN-γ, IL-1β, IL-4, TNF-α) in the serum were evaluated by ELISA. Hematoxylin and eosin (HE) staining was performed to analyze pathological changes, and transmission electron microscopy (TEM) was used to observe ultrastructural changes. The p-p65/p65 protein expression was evaluated by western blotting and the levels of MMP-2 and TGF-β in adhesion tissues were assessed by immunohistochemistry.

**Results:** KFXYS lowered the rectal temperature and the WBC counts in the blood in the acute stage of PID and alleviated inflammatory cell infiltration of the uterus, especially when combined with levofloxacin. KFXYS significantly decreased the levels of proinflammatory cytokines (IFN-γ, IL-1β, IL-4) and adhesion-related factors (TNF-α) and protected the ultrastructure of endometrial epithelial cells. Mechanistically, KFXYS inhibited the NF-κB activation by decreasing phosphorylation of p65, thus the alleviation of inflammation further reduced the expression of TGF-β and MMP-2, and inhibited the occurrence of uterine adhesions.

**Conclusion:** These results revealed that KFXYS alleviated pelvic inflammation and effectively inhibits inflammation-associated adhesion, which indicated the potential role of KFXYS for treatment of PID and the prevention of PID sequelae.

## Introduction

Pelvic inflammatory disease (PID) is infection-induced inflammation of the female upper reproductive tract, and endometritis is an intermediate stage in the pathogenesis of disease (Brunham et al., 2015). The symptoms and signs of PID, which is a major concern of gynecologists, are often very subtle, and delayed diagnosis contributes to long-term reproductive disability, including infertility, ectopic pregnancy, and chronic pelvic pain, which mostly result from inflammatory tissue adhesion (Ness et al., 2002; Revzin et al., 2016). The healthcare cost of PID was previously estimated to be $1,995 per patient, not including expenses related to future evaluation and the treatment of complications (Curry et al., 2019). Therefore, early treatment of acute PID and prevention of pelvic adhesions is the main goal of PID management (Risser et al., 2017). Antibiotics, such as levofloxacin, piroxicam, and fentiazac, can be used to control pelvic inflammation, but the development of antibiotic resistance after long-term use and the pharmacological effects of antibiotics on adhesion need to be further explored (Dhasmana et al., 2014).

Due to the disadvantages of antibiotics and surgical treatment, Traditional Chinese Medicine (TCM) formulations, which can effectively alleviate clinical symptoms, reduce the use of antibacterial drugs and shorten the course of the disease, have become the focus of research on the treatment of PID sequelae and chronic pelvic inflammatory disease (CPID) (Bu et al., 2015; Zhang et al., 2017). Kangfuxiaoyanshuan (KFXYS) is composed of eight classic medicine, namely, *Sophora* flavescens *[*Leguminosae*; Sophorae flavescentis radix* ]*;* Herba Patriniae *[*Caprifoliaceae*;*Patrinia villosa (*Thunb.*) *Dufr.* ]*;* Philippine violet *[*Violaceae*; Viola philippica Cav.*]*;* Andrographis Herba *[*Acanthaceae*; Andrographis paniculata (Burm. F.) Nees* ]*;* Taraxaci Herba *[*Compositae*; Taraxacum mongolicum Hand.-Mazz*]*;*Aloe *[*Asphodelaceae*; Aloe vera (L.) Burm. f.* ]; Lithospermi radix *[*Boraginaceae*; Lithospermum erythrorhizon Siebold&Zucc* ] *and* Suis Fellis Pulvis ***.*** The various chemical components of the compound, which include alkaloids, flavonoids, diterpene lactones, bile acids, anthraquinones, naphthoquinones, and organic acids, were identified in previous studies (C. P. Commission, 2015; Xie et al., 2019). Importantly, pharmacological studies on KFXYS have confirmed its effect in clearing heat, detoxifying, destroying parasites, and relieving itching. Hence, KFXYS has been proven to have good curative effects on chronic adnexitis, chronic pelvic inflammatory disease and vaginitis resulting from perineal incision during delivery (Gao and Zhang, 2017). Previous clinical studies have shown that KFXYS combined with levofloxacin can shorten the course of the disease and reduce the occurrence of PID sequelae. Nevertheless, the pharmacological action and potential mechanism of KFXYS have not been fully elucidated.

Multiple pathogens and multiple infections can enhance inflammation of the upper genital tract, and a rat model of PID reflecting this phenomenon was previously established in our previous research (Fan et al., 2021). In this study, we observed that KFXYS alone or together with antibiotics alleviated PID in rats. We also evaluated the efficacy of different therapeutic strategies in the acute stage of PID and reduced the occurrence of PID sequelae using various methods. Furthermore, we elucidated the mechanism by which KFXYS alleviates acute inflammation and prevents tissue adhesion to determine the effect of KFXYS on PID.

## Materials and methods

### Preparation of the KFXYS and levofloxacin tablets

KFXYS (batch No. 20190603) was provided by Sunflower Pharmaceutical Group Co., Ltd., and levofloxacin tablets were purchased from Beijing Obstetrics and Gynecology Hospital, Capital Medical University. The weights and ratios of various drugs are shown in Table 1.The preparation of KFXYS are as follows: in addition to Suis Fellis Pulvis and Aloe*,* the other six kinds of herbal medicine were decocted with water for two times: 2 and 1.5 h. The liquid was filtered and dried below 70°C, and was crushed into fine powder. Suis Fellis Pulvis and Aloe were directly dried and ground into powder. Then 966.7–1,015.3 g fatty acid glyceride was prepared and dissolved below 45 °C, and mixed with the above powder. Stir and mix all ingredients for 30 min, and pour mould at 40–45 °C. Each grain weighs 2.0g, and the weight difference limit is ±5%.

**TABLE 1:** The ratio of the components present in the preparation of Kangfuxiaoyanshuan (KFXYS).

Components	Weight (g)	Amount in application (ratio)
S (%)ophora flavescens *[*Leguminosae*; Sophorae flavescentis radix*]	690	9.30
Herba Patriniae *[*Caprifoliaceae*;*Patrinia villosa (*Thunb.*) *Dufr.* ]	1,150	15.49
Philippine violet *[*Violaceae*; Viola philippica Cav.*]	920	12.39
Andrographis Herba *[*Acanthaceae*; Andrographis paniculata (Burm. F.) Nees*]	1,150	15.49
Taraxaci Herba *[*Compositae*; Taraxacum mongolicum Hand.-Mazz*]	2,230	30.04
Suis Fellis Pulvi	100	1.35
Lithospermi radix *[*Boraginaceae*; Lithospermum erythrorhizon Siebold&Zucc*]	1,145	15.43
Aloe *[*Asphodelaceae*; Aloe vera (L.) Burm.f.*]	38	0.51

Four gram of KFXYS was diluted in 10.0 ml of physiological saline and dissolved by heating to obtain 400 mg/ml KFXYS solution. Then, 4 g levofloxacin was diluted in 80 ml physiological saline to obtain 50 mg/ml levofloxacin solution. All drug formulations were prepared every 4 days and stored at 2–8°C. The final dosage of KFXYS was 400 mg/kg/day, and the levofloxacin was 50 mg/kg/day.

### Microorganisms

*Staphylococcus(S.) aureus* (ATCC25923) and *Escherichia(E.) coli* (ATCC25922) were used in this study. Prior to the experiment, each bacteria was collected in the logarithmic growth phase and adjusted the concentration to 3 × 10^9^CFU/ml. 1 h before the experiment, the two bacteria solution were mixed in a ratio of 1:1 with a final concentration of 3 × 10^9^ CFU/ml. During the modeling experiment, each horn of uterus was inoculated with 0.1 ml bacterial suspension, that is, the inoculated amount of 6 × 10^8^CFU bacteria for each rat.

### Rat model of PID

Estrus was identified by vaginal smear for two continuous cycles, and only rats with regular cycles were included in the subsequent experiments. Suitable mice were randomly allocated into a sham operation group (*n* = 15) and an experimental PID model group (*n* = 60). A mouse model of PID was established as we previously reported (Fan et al., 2021). Briefly, the rats were fasted overnight and then anesthetized with an intraperitoneal injection of 1% pentobarbital sodium (30 mg/kg). The uterus was exposed by making a 0.8–1 cm incision along the midline of the lower abdomen, and the uterus was exposed and securely positioned after laparotomy. Then, a hypodermic needle containing 0.2 ml of bacterial suspension was gently inserted longitudinally into the uterine horn in the direction of the fallopian tube. The endometrium was slightly damaged by gentles wabbing with pillow, and then 0.1 ml bacterial solution was injected into the lumen of the uterine horn. The contralateral uterine horn was subjected to the same protocol, and then the abdominal cavity was closed sutured. The abdomen of sham group rats was opened and closed immediately, with no manipulation of the uterus.

### Experimental design and study groups

In this study, the day of model established was considered D-3. Three days after infection (D0), 60 successfully infected mice were randomly divided into the following four groups (*n* = 15): the PID model group (untreated), Levofloxacin group (levofloxacin, 50 mg/kg/day), KFXYS group (KFXYS, 400 mg/kg/day), and KFXYS + Levofloxacin group (KFXYS, 400 mg/kg/day + levofloxacin, 50 mg/kg/day). The levofloxacini administrated by intragastric algavage and KFXYS is administrated rectally. All drug treatments lasted 14 days (D0-D14). The rectal temperature of the rats was measured, and blood samples were collected from the tail vein every 2 days from D0-D14 for routine blood tests. Samples were collected at D3, D14, and D49. After isoflurane anesthesia, 2 ml blood was taken from the eyeball for subsequent ELISA, and after laparotomy, the left uterus, ovaries and fallopian tubes were fixed for immunohistochemistry and transmission electron microscopy (TEM). Tissues from the other side were flash frozen in liquid nitrogen.

### Enzyme-linked immunosorbent assay

QuantiCyto^®^ ELISA kits for IL-1β (Cat No. ERC007), IL-4 (Cat No. ERC002), IFN-γ (Cat No. ERC101 g), and TNF-α (Cat No. ERC102a) were purchased from Neobioscience Biotechnology Co., Ltd. (Beijing, China), and the experimental procedures were conducted according to the manufacturer’s instructions. Briefly, 100 μl rat serum was added to each coated well and incubated with antibody at 37°C for 2 h. Then, the wells were washed with PBS. Then, biotinylated antibody was added and incubated for 1 h. After washing with PBS, chromogenic substrate was added, and 100 μl of 2 M sulfuric acid was added to terminate the reaction. Finally, the OD value at 450 nm was measured using a microplate reader, and the concentrations of proteins of interest the samples were calculated according to a standard curve.

### Hematoxylin and eosin staining and analysis

The harvested uterine tissues were fixed in 10% formaldehyde solution, dehydrated through analcohol gradient, embedded in paraffin wax and cut into 4-μm thick sections with a microtome. Before staining, the sections were dewaxed in xylene, rehydrated through decreasing concentrations of ethanol, and washed with PBS. Then, the sections were subjected to HE staining and examined by light microscopy.

### Immunohistochemistry

Tissue sections were prepared as previously described. The prepared slides were first subjected to antigen repair with 0.01 M citric acid buffer for 15–20 min at 95°C. Following washing with PBS, the slides were incubated with polyclonal antibody diluted in serum overnight at 4°C.The next day, the slides were incubated with a horseradish peroxidase (HRP)-conjugated secondary antibody (1:100) at room temperature for 1 h, stained with diaminobenzidine (DAB), and counterstained with hematoxylin. The following antibodies were used: TGF-β polyclonal antibody (1:100, cat number 21898-1-AP, Proteintech), MMP-2 polyclonal antibody (1:100, cat number 10373-2-AP, Proteintech), and IgG (Zhongshan Golden Bridge Biotechnology). The immunostained tissue sections were examined by light microscopy and analyzed.

Semiquantitative analysis was performed using histochemistry score (H-score), which is a histological scoring method for immunohistochemistry. H-scores range from 0 to 300, with a higher score indicating stronger staining.

### Western blotting analysis

Uterine tissues were lysed using RIPA lysis buffer containing1 mM PMSF. The concentration of the extracted proteins was quantified using a BCA Protein Assay Kit (Beyotime, China), and the proteins were denatured by boiling for 10 min. Fifteen micrograms of protein was separated on a 10% sodium dodecyl sulfate (SDS)-polyacrylamide gel and then transferred onto a PVDF membrane (0.22 μm, Millipore) by electroblotting. The membrane was blocked in 5% free-fat milk in TBST for 1 h at room temperature and then incubated with primary antibodies at 4°C overnight. The following antibodies were used in this study: p-p65 (1:1,000; cat number 3033, Cell Signaling Technology), p65 (1:1,000; cat number GB121142, Servicebio), MMP-2 (1:800; cat number 10373-2-AP, Proteintech) and β-actin (1:2000; cat number GB15001, Servicebio). On the second day, the membrane was washed and incubated with appropriate HRP-conjugated secondary antibodies. The immunoreactive bands were visualized using chemiluminescence reagent and images using a chemiluminescence image analysis system (Tanon, Tanon 5,200, China). Relative protein levels were normalized to β-actin levels and then compared with the expression levels in the control group, and Alpha Ease FC software was used to semiquantify protein expression.

### Transmission electron microscopy

Two-millimeter-thick uterine tissues were placed in 3% glutaraldehyde and 1% osmium tetroxide for at least 24 h at 4°C as previously reported. The tissues were then dehydrated, embedded and cut into 70-nm thick sections. Finally, the samples were dried with a HitachiES-2030 dryer, sprayed with gold with a HitachiE-1010 injector, and observed with a Hitachi HT770 transmission electron microscope. Changes in organelles in endometrial cells were observed at different magnifications, and additional morphological features of the epithelia were observed independently by two investigators.

### Statistical analysis

GraphPad Prism 5.0 and SPSS Statistics 22.0 software were utilized for plotting and analysis of all data. All data were confirmed by over three repeated trails and were shown as means ± standard deviation (SD) or means ± standard error of mean (SEM). Data conforming to normal distribution were evaluated with a 2-tailed unpaired Student’s t test and the comparisons between multiple groups were performed by one-way ANOVA followed by Dunnett’s multiple comparison test. *p* < 0.05 was considered statistically significant, as indicated by asterisks (*p < 0.05, **p < 0.01, ***p < 0.001). β-actin was used as an internal control western blot analysis.

## Results

### Ameliorating effects of KFXYS on inflammation in the acute stage of PID in rats

Kangfuxiaoyanshuan (KFXYS) is composed of eight classic medicinal, namely, *Sophora* flavescens *[*Leguminosae*; Sophorae flavescentis radix* ]*;* Herba Patriniae *[*Caprifoliaceae*;*Patrinia villosa (*Thunb.*) *Dufr.* ]*;* Philippine violet *[*Violaceae*; Viola philippica Cav.*]*;* Andrographis Herba *[*Acanthaceae*; Andrographis paniculata (Burm. F.) Nees* ]*;* Taraxaci Herba *[*Compositae*; Taraxacum mongolicum Hand.-Mazz*]*;*Aloe *[*Asphodelaceae*; Aloe vera (L.) Burm. f.* ]; Lithospermi radix *[*Boraginaceae*; Lithospermum erythrorhizon Siebold&Zucc* ] *and* Suis Fellis Pulvis. The weight and ratio of the components in the preparation were presented in Table 1.

**FIGURE 1:**
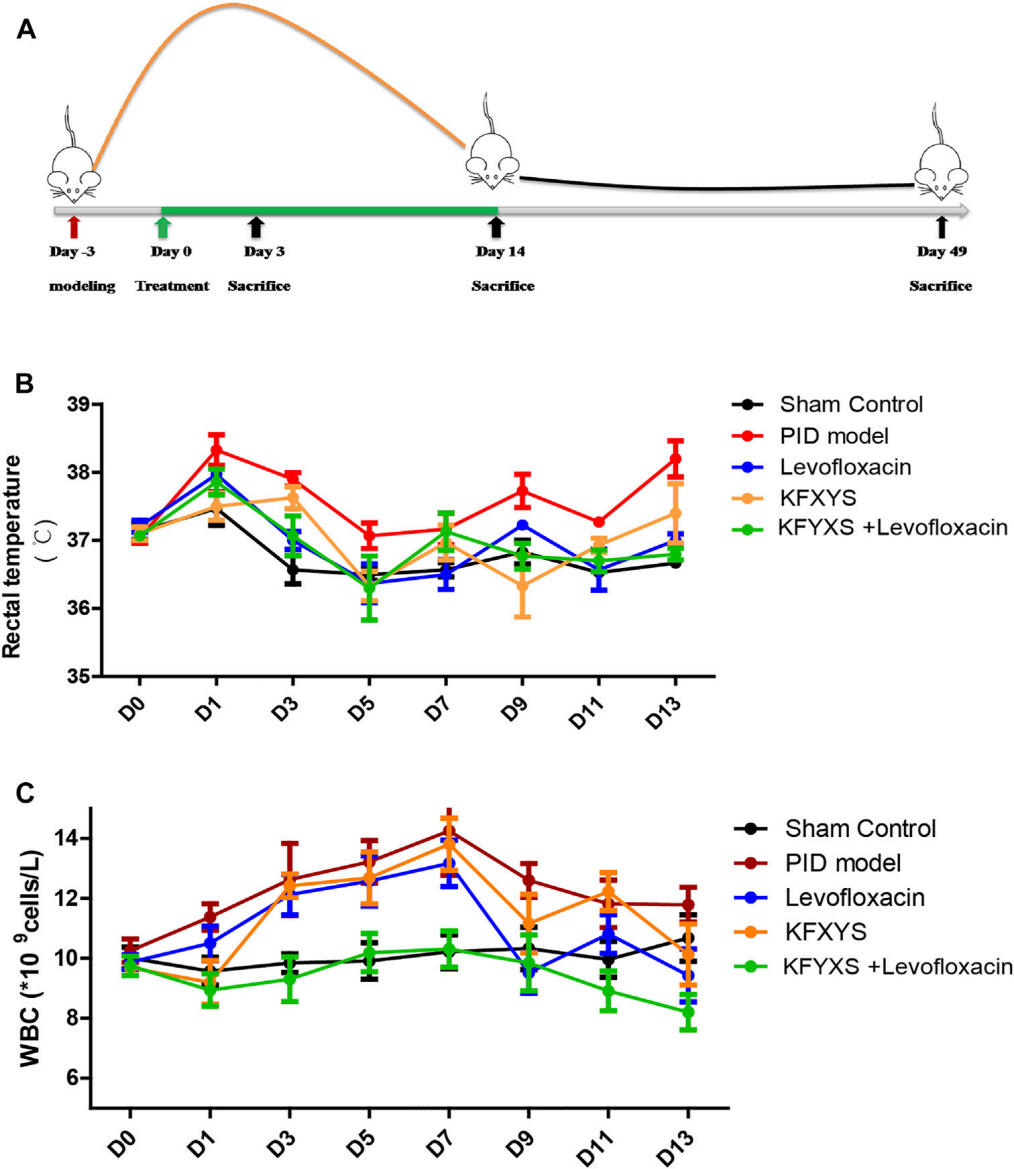
**Schematic diagram of the experiment and changes in rat rectal temperature and WBC counts after treatment with different drugs.** **(A)** Schematic diagram of the experimental setup. The first treatment day was denoted as D0.At D3, rats received 6 × 10^8^CFU *E. coli* and *S. aureus* solutions and underwent endometrial scratching. Treatment continued for 14 days, and tissues were sampled at D3, D14 and D49. **(B)** The changes in the rectal temperature of the rats during 14 days of treatment. The mean ± SEM are plotted in the graph; *n* = 4. **(C)** The infection indices and WBC counts determined from samples taken from the eyeballs of the rats during 14 days of treatment. The mean ± SEM are plotted in the graph; *n* = 7. Sham control: rats received laparotomy without manipulation and treatment. PID model: rats were untreated following the PID operation. Levofloxacin: rats that received 50 mg/kg/day levofloxacin orally for 14 days following the operation. KFXYS: rats that received 400mg/kg/day KFXYS rectally for 14 days following the operation. KFXYS + Levofloxacin: rats that received 400mg/kg/day KFXYS rectally and 50 mg/kg/day levofloxacin orally for 14 days following the operation.

According to our previous study, PID model rats experience a 14- to 21-day period of acute inflammation, with the degree of inflammation being the highest at 6–8 days, followed by a period of chronic inflammation and sequelae with adhesion (Fan et al., 2021). The day of model establishment was considered D-3, drug was administered from D0 to D14, and samples were collected on D3, D14, and D49 (Figure 1A).

After the model was established, the rats developed a high fever due to bacterial infection of the pelvic cavity. The rectal temperature of the sham operation group increased temporarily due to opening of the abdomen. Severe pelvic infection caused an increase in rectal temperature in the PID model rats. Although the rectal temperature was significantly decreased during treatment with levofloxacin (D3, D13) or KFXYS (D9), the combination treatment group showed a greater reduction in rectal temperature, especially at D9 and D13 (Figure 1B; Table 2).

**TABLE 2:** Changes of rectal temperature of rats in acute inflammatory stage (*n* = 4, ‾X ± sem, °C).

Rectal temperature	Sham control	PID model	Levofloxacin	KFXYS	KFXYS + levofloxacin
D0	37.13 ± 0.117	37.04 ± 0.079	37.21 ± 0.089	37.10 ± 0.097	37.07 ± 0.069
D1	37.47 ± 0.247*	38.33 ± 0.225	37.97 ± 0.029	37.50 ± 0.200	37.86 ± 0.189
D3	36.57 ± 0.208 ***	37.90 ± 0.100	37.00 ± 0.132*	37.63 ± 0.161	37.07 ± 0.290
D5	36.50 ± 0.087	37.07 ± 0.189	36.37 ± 0.284	36.33 ± 0.214	36.30 ± 0.471
D7	36.57 ± 0.104	37.17 ± 0.231	36.50 ± 0.218	36.97 ± 0.257	37.13 ± 0.276
D9	36.83 ± 0.176*	37.73 ± 0.246	37.23 ± 0.076	36.33 ± 0.454 ***	36.77 ± 0.189*
D11	36.53 ± 0.076	37.27 ± 0.076	36.57 ± 0.301	36.93 ± 0.104	36.70 ± 0.158
D13	36.67 ± 0.076 ***	38.20 ± 0.265	37.00 ± 0.100 ***	37.40 ± 0.436	36.80 ± 0.087 ***

*p< 0.05 vs*.* the PID, model group, **p< 0.01vs*.* the PID, model group, and ***p< 0.001 vs*.* the PID, model group.

We also monitored white blood cell (WBC) counts in the blood, which is an indicator of infection-related inflammation. Previous study showed a temporary decrease in WBC in PID model group rats at the beginning of 0–2 days, which might be caused by the severe infection in PID rats (Fan et al., 2021). The WBC count in the PID model group remained elevated until D7 and then began to decline, but it was still at a high level. Levofloxacin alone has a certain effect on WBC reduction (D9, *p* < 0.05), while the reduction in the WBC count was more pronounced in the combination treatment group, in which the WBC count returned almost to normal levels (Figure 1C;Table 3).

**TABLE 3:** Changes of white blood cell (WBC) of rats in acute inflammatory stage [n = 7, ‾X ± sem,*10^9^cells/L)].

WBC	Sham control	PID model	Levofloxacin	KFXYS	KFXYS + levofloxacin
D0	10.01 ± 0.357	10.26 ± 0.391	9.87 ± 0.247	9.69 ± 0.175	9.75 ± 0.32
D1	9.57 ± 0.461	11.38 ± 0.442	10.5 ± 0.581	9.20 ± 0.729	8.94 ± 0.547
D3	9.84 ± 0.311*	12.63 ± 1.204	12.13 ± 0.678	12.42 ± 0.391	9.30 ± 0.749 **
D5	9.91 ± 0.609 **	13.22 ± 0.709	12.58 ± 0.842	12.69 ± 0.868	10.19 ± 0.637*
D7	10.22 ± 0.568***	14.25 ± 1.479	13.17 ± 0.772	13.81 ± 0.873	10.31 ± 0.606 ***
D9	10.33 ± 0.710	12.60 ± 0.566	9.53 ± 0.687*	11.16 ± 0.974	9.85 ± 0.930
D11	9.96 ± 0.595	11.82 ± 0.794	10.81 ± 0.650	12.23 ± 0.633	8.92 ± 0.665*
D13	10.68 ± 0.777	11.79 ± 0.578	9.43 ± 0.885	10.13 ± 1.014	8.20 ± 0.592 **

*p< 0.05 vs*.* the PID, model group, **p< 0.01vs*.* the PID, model group, and ***p< 0.001 vs*.* the PID, model group.

### Comparison of the anti-inflammatory effects of different drugs in the acute stage of PID

**FIGURE 2:**
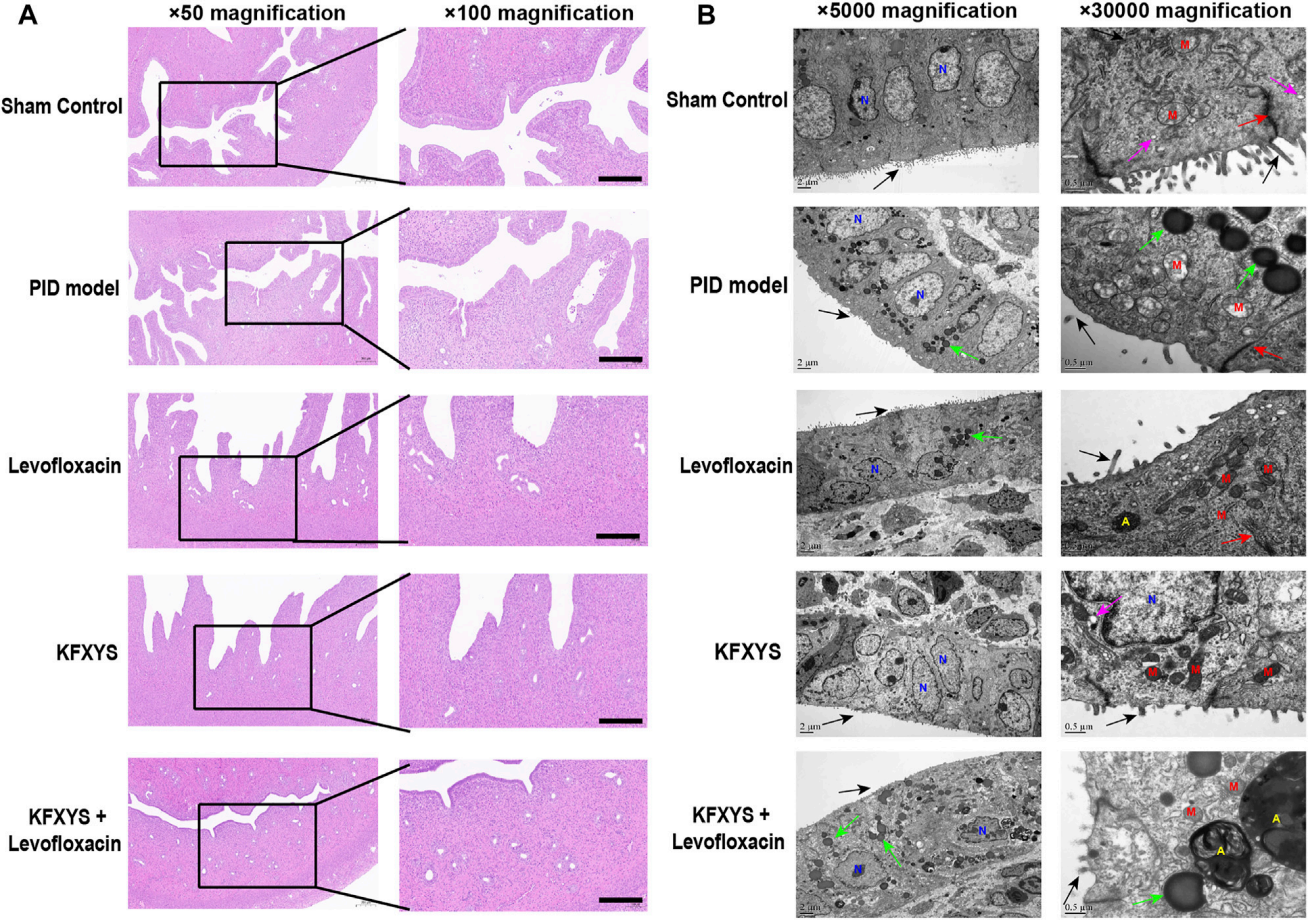
**Inflammatory changes in uterine tissue associated with drug treatment during the acute inflammation phase (D3).** **(A)** Changes in inflammatory cells in the uterine mucosa were observed by HE staining. In the PID model group, the epithelial cells were hyperplastic and dense, showing papillary protrusion into the uterine cavity, some interstitial structures were destroyed, and a large number of chronic inflammatory cells were observed in the mucosal layer and muscle layer. In the Levofloxacin or KFXYS group, the formation of papillary protrusions by epithelial cell proliferation was reduced, and there was a small number of inflammatory cells and infiltrating neutrophils in the mucosal layer. There were almost no inflammatory cells and epithelial and glandular structures were intact in the KFXYS+Levofloxacin group. Images were taken at 50× or 100×; magnification. Scale bar: 100 μm. **(B)** Ultrastructural changes in the uterine mucosa of rats observed by TEM. Intact microvilli (black arrows), intercellular connections (red arrows), secretory vesicles (purple arrows), and lipid droplets (green arrows) were observed. N, nucleus; M, mitochondrion; A, autolysosome. Images were taken at 5000× or 30000× magnification.

We assessed pelvic inflammation in rats on D3, which was the seventh day after the establishment of the model, and the duration of acute inflammation in the pelvic cavity. The abdominal cavity of the rats was opened to observe inflammation in the pelvic cavity, and then the tissues were subjected to HE staining. In the sham group, epithelial tissues and glands were intact and showed no inflammatory cell infiltration. However, in the model group, epithelial cells were hyperplastic and showed papillary protrusion into the uterine cavity, some interstitial structures were destroyed, the cells were disorganized, and a large number of chronic inflammatory cells (lymphocytes, monocytes, plasma cells) and neutrophils were observed in the mucosal layer and muscle layer. Inflammatory cells were seen only in the superficial layer of the endometrium after antibiotic treatment, and KFXYS also partly reduced inflammation of the uterus. Notably, epithelial and glandular structures were restored after combined treatment with levofloxacin and KFXYS, and almost no inflammatory cells were seen in the mucosal layer of the endometrium (Figure 2A).

We also performed TEM to observe ultrastructural changes to assess the probiotic properties of the different drugs (Figure 2B). The mucosa of the sham control tissue appeared normal, with no signs of inflammation and many secretory vesicles. A number of slender microvilli and glycocalyx covered the single columnar epithelium, and the intercellular connective complex and mitochondria were normal. In the pelvic inflammation model group, the number of microvilli on the surface was significantly reduced, the calyx disappeared, and with significant intracellular lipid degeneration, severe mitochondrial swelling, and even mitochondrial rupture. The number of microvilli on the cell surface and the morphology of mitochondria were improved after levofloxacin treatment, but many lipid droplets were still visible. Although KFXYS treatment effectively restored the morphology of mitochondria, the intracellular spaces were large, desmosomal junctions were significantly impaired and blurred, and there was interstitial cell disorder. Fortunately, microvilli were restored and intracellular mitochondrial swelling were notably alleviated in the KFXYS + levofloxacin group. There were still some lipid droplets, but autophagosomes were formed, indicating that a protective mechanism was activated in the cells to minimize the damage caused by inflammation. These observations indicated that KFXYS has a certain effect on controlling inflammation in the acute stage of PID, especially when combined with levofloxacin.

### Changes in the levels of immune- and adhesion-related factors after treatment with different drugs

**FIGURE 3:**
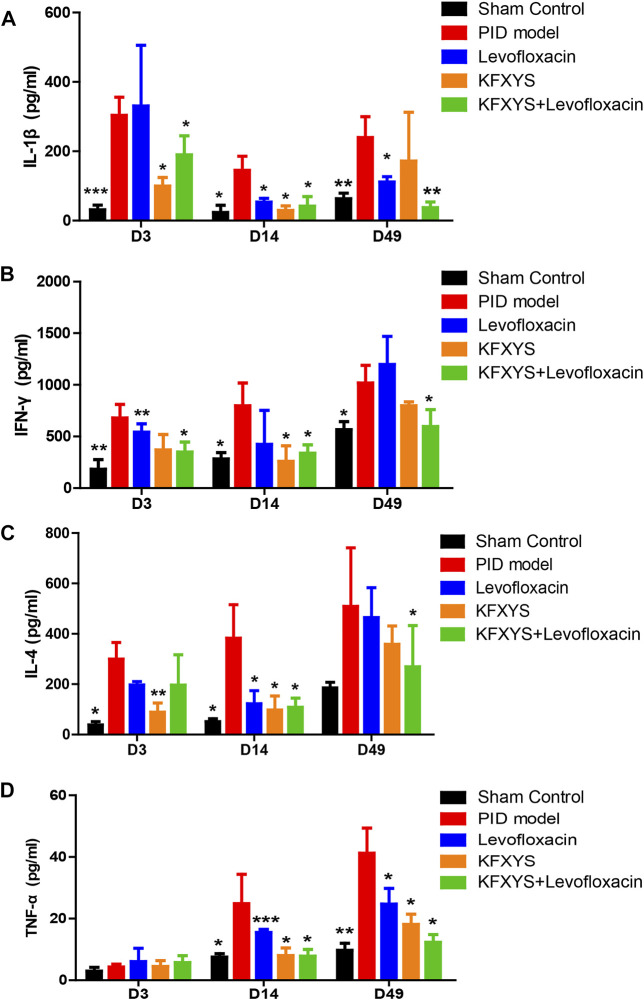
**Decreasing effect of different drugs on the blood levels of the immune-related factors IL-1β, IFN-γ, IL-4, and TNF-α in rats.** Expression changes are presented as the mean ± SD (*n* = 4). The data were analyzed using one-way ANOVA and unpaired t test. *p < 0.05 vs the PID model group, **p < 0.01 vs the PID model group, and *** *p* < 0.001 vs the PID model group.

The rats were sacrificed at D3, D14 and D49, and the serum levels of the immune factors IL-1β, IL-4, IFN-γ and TNF-α were analyzed by ELISA. IL-1β, which is mainly secreted by macrophages, B cells, and dendritic cells, has proinflammatory activity and leads to extensive inflammatory events, initiating the antibacterial inflammatory response of the organism. In the acute stage of PID (D3), IL-1β levels were markedly increased, indicating severe inflammation. Il-1βexpression was decreased after treatment with levofloxacin or KFXYS, and the combination treatment resulted in a greater degree of reduction in IL-1β levels (Figure 3A).

Similarly, IFN-γ is an important inflammatory regulator and a hallmark cytokine of Th1 and natural killer cells. In this study, varying decreases in the expression levels of IFN-γ were observed on D3 and D14 after treatment with different drugs, but the difference was only significant in the combined group at D49 (Figure 3B).

IL-4 is a pivotal immune modulator for adaptive immune regulation and is mainly secreted by Th2 cells, which is involved in various inflammatory pathophysiological processes. Strikingly, decreased production of IL-4, which was mainly observed in the early stage, may underlie the alleviation of edema and exudation of uterine tissue after treatment with different drugs (Figure 3C).

As an important inflammatory cytokine, TNF-α is closely related to the extent of intraperitoneal adhesion. We observed a low level of TNF-α in the early stage of PID and a gradual increase in TNF-α expression in the PID model group, mainly due to the emergence and aggravation of interstitial adhesion. Fourteen days of levofloxacin or KFXYS treatment alone effectively reduced TNF-α expression, but combined use lowered TNF-α serum levels and better prevented tissue adhesion, which is consistent with the histopathological observations (Figure 3D).

### Histopathological and ultrastructural changes in uterine tissue after 14 days of treatment and the expression of p65 and p-p65

**FIGURE 4:**
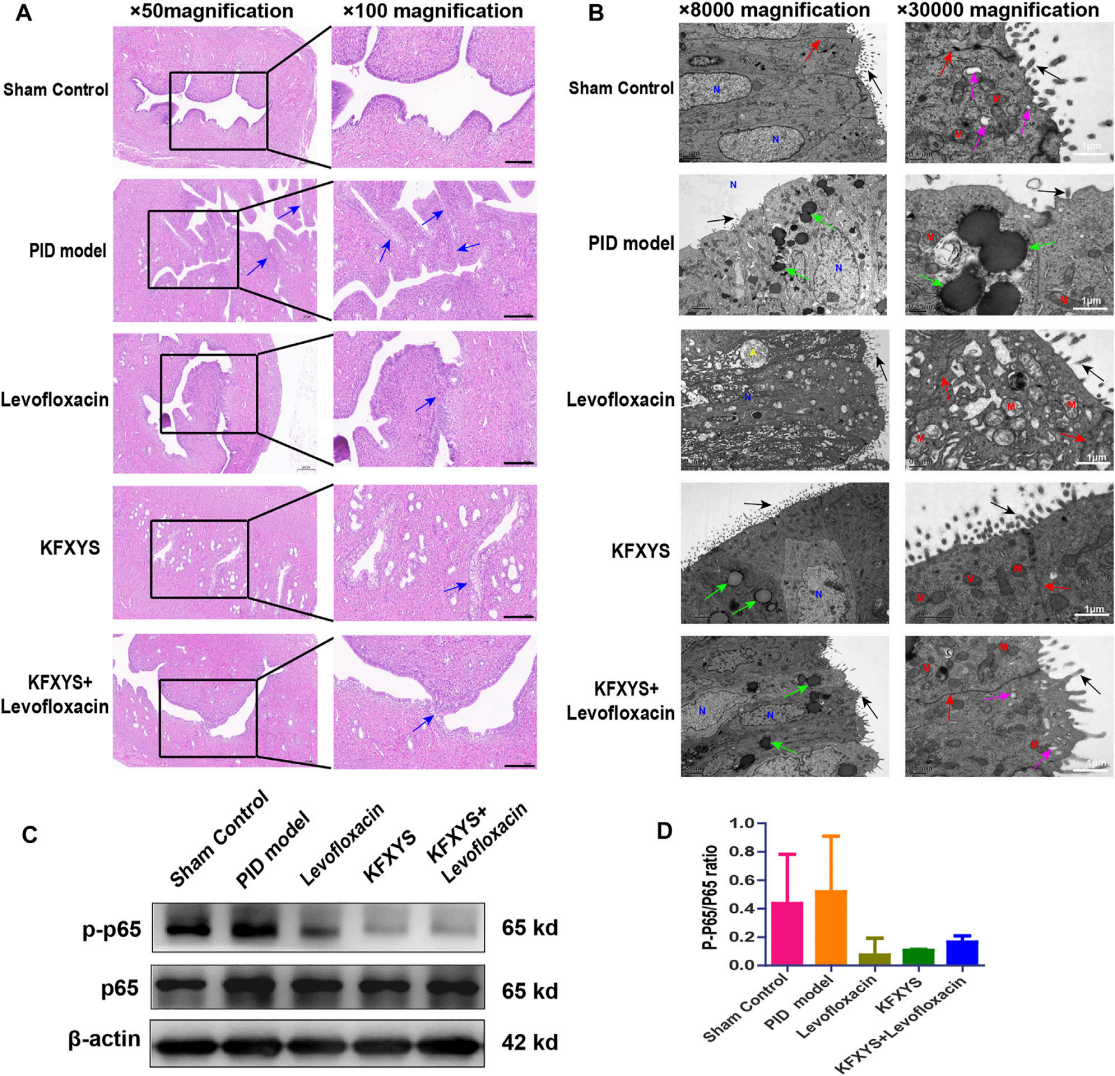
**Histopathological and ultrastructural changes in uterine tissue after 14 days of treatment and the expression of p65 and p-p65.** **(A)** Uterine mucosa adhesion was observed by HE staining. The black arrows indicate bands of adhesive tissue. Images were taken at 50× or 100× magnification. Scale bar: 200 μm. **(B)** Ultrastructural changes in the uterine mucosa of rats observed by TEM. Intact microvilli (black arrows), intercellular connections (red arrows), secretory vesicles (purple arrows), and lipid droplets (green arrows) were observed. N, nucleus; M, mitochondrion; A, autolysosome. Images were taken at 5000× or 30000× magnifications. Scale bar: 1 μm. **(C)** Western blot analysis was used to measure the expression levels of p65 and p-p65 at D14.β-actin was used as an internal control. **(D)** The ratio of p-p65/p65 protein levels from three independent western blot analyses.

Persistent pelvic inflammation causes tissue adhesions. In our previous study, it was found that tissue adhesions began to appear approximately 14 days after model establishment. Similarly, HE staining was used to evaluate the preventive effect of different drugs. Unexpectedly, there were extensive and obvious adhesions in the endometrium layer at D14 in the model group, with increased papillary protrusions in the uterine mucosa. Levofloxacin or KFXYS treatment for 14 days had a certain effect on the adhesion reduction, but the combination treatment better protected the uterine mucous membrane and alleviated uterine mucosa adhesion (Figure 4A).

The ultrastructural changes in the endometrium were also analyzed by TEM. As shown in Figure 4B, the endometrium of the controlgroup showed anormal shape, with rich microvilli and mitochondria with normal morphology. In the pelvic inflammation model group, cell morphology was irregular, the number of microvilli on the surface was decreased, and the calyx disappeared. Furthermore, there were a large number of intracellular lipid droplets, mitochondria were mildly to severely swollen, the matrix density was decreased, and crista fracture or disordered arrangement was observed. After levofloxacin treatment, the microvilli and intercellular space of epithelial cells were restored. However, the overall arrangement of cells was disordered, matrix density was decreased, and there were many vacuoles. Additionally, obvious mitochondrial swelling and intracellular cytolysosomes were observed. KFXYS partially restored the intercellular space and microvilli on the cell surface; however, mitochondria were slightly swollen in the cytoplasm, and some liposomes were still present. Fortunately, the slender microvilli and intercellular space were partly restored after treatment with the combination of levofloxacin and KFXYS. Although there were some droplets present, secretory vesicles reformed in the endometrium, suggesting that the ultrastructure was almost completely restored.

Nuclear factor-κB (NF-κB) is an important intracellular nuclear transcription factor, which participate in the body’s inflammatory response, immune response, and stress response. In our study, the level of NF-κB (p65) of uterine at D14 was detected by western blotting (Figure 4C). The expression of p65 increased sharply in PID rat and accompanied by an increase in p-p65 expression, indicating the activation of the NF-κB signaling pathway. Treatment with levofloxacin or KFXYS effectively decreased the expression and phosphorylation of p65, and even reduced the p-p65/p65 ratio, which represents the inhibition of the NF-κB pathway (Figure 4D). Therefore, these results suggested that the anti-inflammatory effect of KFXYS may be through inhibition of changes in p-p65/p65 content.

### Effect of different drugs on the expression levels of TGF-β at D14 and D49

**FIGURE 5:**
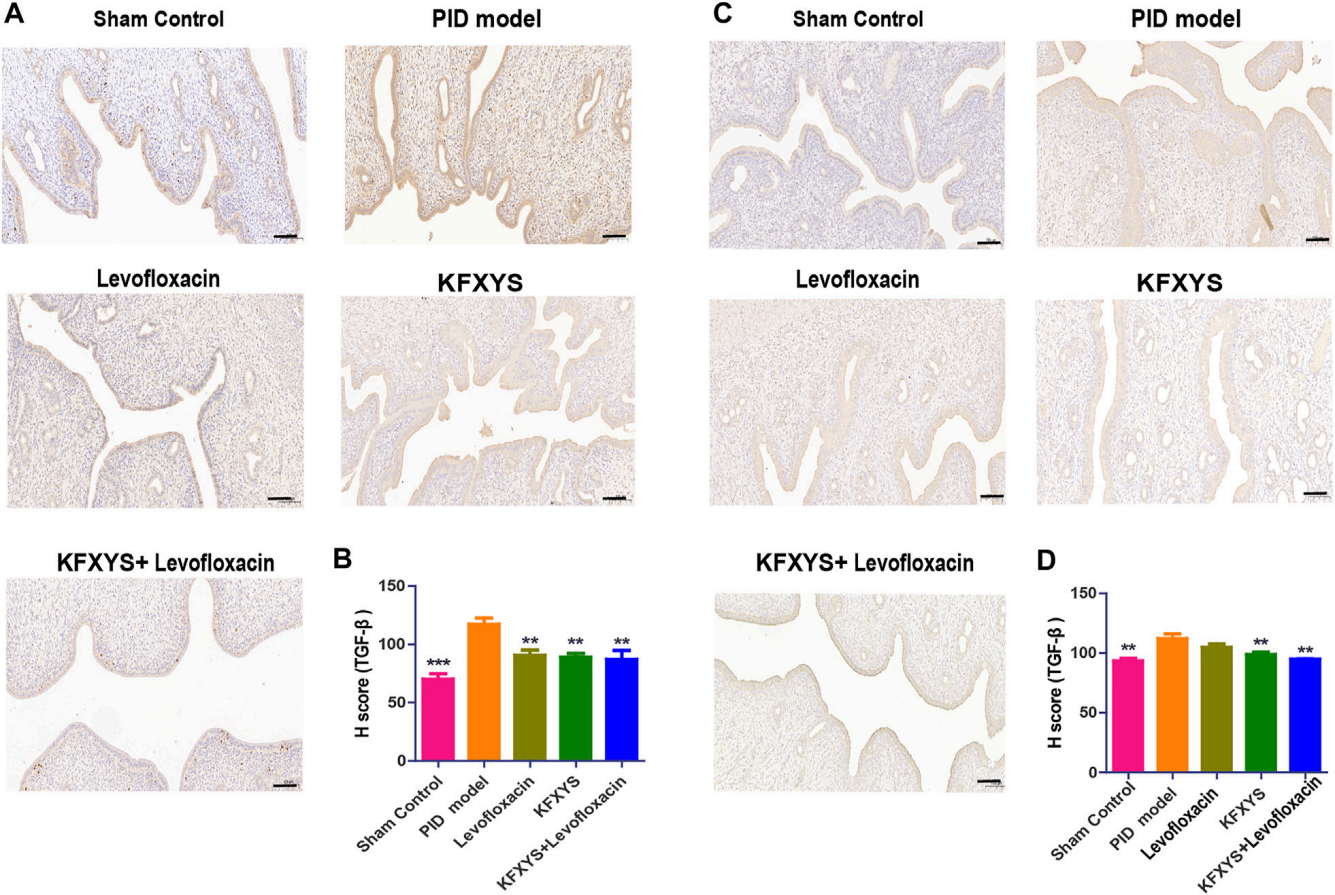
**The expression of TGF-β at D14 and D49 by immunohistochemistry analysis.** **(A)** Immunohistochemistry was used to test the protein expression of TGF-β in adhesive uterine tissue at D14. **(B)** Semiquantitative analysis by the H-score of TGF-β at D14. **(C)** The protein expression of TGF-β in adhesive uterine tissue at D49. **(D)** Semiquantitative analysis by the H-score of TGF-β at D49 Scale bar: 100 μm. **p < 0.01 vs the PID model group, and *** *p* < 0.001 vs the PID model group.

To explore the mechanism of different drugs against adhesion, we detected the expression level of TGF-β, which is closely associated with the occurrence and development of intrauterine adhesion. The expression of TGF-β at D14 and D49 was detected by immunohistochemistry and quantified by H-Score analysis. Pathological results showed that TGF-β expression was significantly up-regulated in PID rats at D14, which may accelerate the occurrence of tissue adhesion and different drug treatments had a certain inhibitory effect on TGF-β expression (Figures 5A,B). Same results were also seen at D49, suggesting that KFXYS treatment may inhibit TGF-β expression (Figures 5C,D).

### Effect of different drugs on reducing tissue adhesion and MMP-2 level in the sequelae stage

**FIGURE 6:**
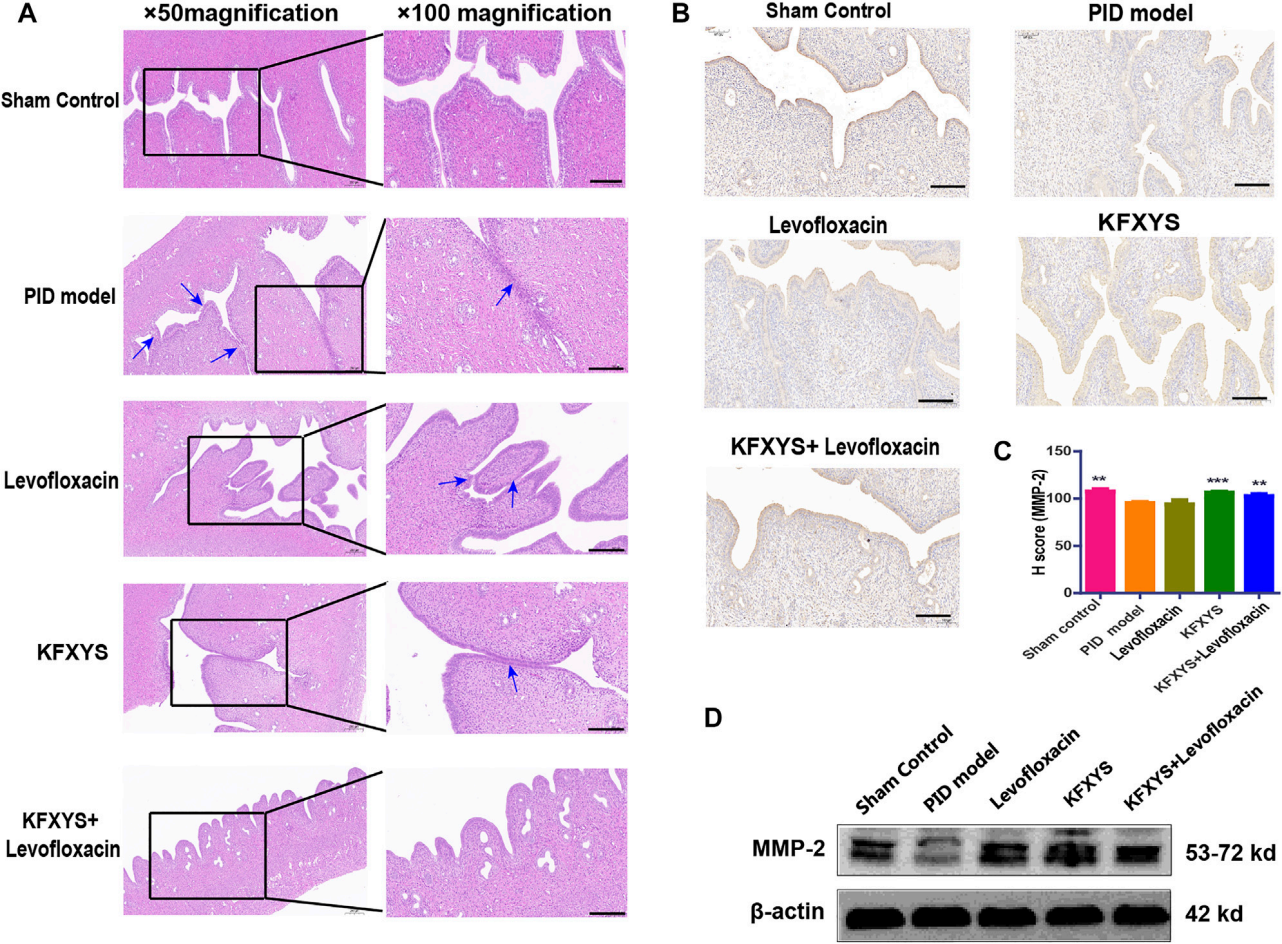
**Histopathological images showing the degree of adhesion of uterine tissue at D49 and analysis of MMP-2 expression by immunohistochemistry.** **(A)** Uterine mucosa adhesion was observed by HE staining. The black arrows indicate bands of adhesive tissue. Images were taken at 50× or 100× magnification. **(B)** Immunohistochemistry was used to analyze the protein expression of MMP-2 in adhesive uterine tissue from the different groups. **(C)** Semiquantitative analysis by the H-score of MMP-2. **(D)** Western blot analysis was used to measure the expression levels of MMP-2 at D49. β-actin was used as an internal control. Scale bar: 200 μm. **p < 0.01 vs the PID model group, and *** *p* < 0.001 vs the PID model group.

The sequela phase of PID begins at D49, and this phase is characterized by persistent pelvic tissue inflammation and subsequent adhesions. Adhesions between uterine tissues and other pelvic tissues were observed in the model group after laparotomy, and HE staining was used to evaluate the adhesions in detail. In the PID model group, the endometrium was showed severe adhesion. Although the adhesion area of the rats treated with levofloxacin or KFXYS was decreased, some of the uterine tissue adhesion was still visible. Intriguingly, in the combined treatment group, papillary protrusion occurred, but the laminae propria mucosae showed almost no adhesion, and uterine wall morphology returned to normal (Figure 6A).

It has been reported that, uterine adhesion is closely related to abnormal accumulation of extracellular matrix (ECM), and matrix metallopeptidase-2 (MMP-2) has a pivotal role in ECM degradation. Although MMP-2 has been indicated to have a crucial role in fibrogenesis, its pattern of expression in fibrotic tissues remains controversial. The results of the present study indicated that MMP-2 was significantly down-regulated in the rat of PID, while the treatment of KFXYS or KFXYS + levofloxacin induced obvious upregulation, thus facilitating ECM degradation and inhibiting the tissue adhesion formation (Figures 6B–D).

## Discussion

PID is usually the result of infection with various microbes that ascend from the endocervix, causing endometritis, salpingitis, and pelvic peritonitis (Tokmak et al., 2017). According to the severity of inflammation and disease course, PID can be divided into acute pelvic inflammation, the main symptoms of which are high fever, lower abdomen pain and abnormal cervical secretions, and Chronic Pelvic Inflammatory Disease (Charvériat and Fritel, 2019). Currently, the Centers for Disease Control and Prevention (CDC) recommends the use of broad-spectrum combination levofloxacin therapies for the treatment of PID to combat the likely pathogens (Centers for Disease Control and Prevention, 2010). Although more than 90% of patients with PID have a clinical response to the CDC-recommended treatment, long-term antibiotic treatment can have obvious adverse effects on the body, mainly involving gastrointestinal injury, weakened immunity, and drug resistance (Crossman, 2006; Baron et al., 2013).

In recent years, as Traditional Chinese Medicine (TCM) have received great attention for their ability to maintain health and control disease, some TCM compounds have been used in the treatment of PID (Bu et al., 2015; Zhang et al., 2017; Jiang et al., 2019; Wang et al., 2019). Researchers proved that Man-Pen-Fang has a significant effect against CPID, probably due to its ability to inhibit the inflammatory response by promoting the apoptosis of inflammatory cells and reducing the expression of inflammatory cytokine in the serum (Zhang et al., 2017). As in past studies, the clinical drug KFXYS, which is normally delivered rectally, was used to treat rats with PID in our study, and the results showed that KFXYS had a certain anti-inflammatory effect and reduced adhesion in the sequelae phase of PID.

An increased number of plasma cells and neutrophils upon transcervical endometrial aspiration is commonly used to confirm the diagnosis of acute PID (Kiviat et al., 1990). Pathological staining of the uterus of rats with PID revealed a large number of chronic inflammatory cells (lymphocytes, monocytes, plasma cells) and neutrophils in the lamina and muscularum of the mucosa. The infiltration of lamina propria cells and neutrophils decreased significantly after treatment with levofloxacin or KFXYS alone, and obvious alleviation of infiltration was observed after treatment with levofloxacin combined with KFXYS. In the acute phase of PID, uterus and pelvic tissue inflammation as well as systemic manifestations, such as fever and increased leukocyte levels, are observed. As KFXYS enhanced the ability of levofloxacin to control inflammation and alleviated infection and high fever in rats to a greater extent, it showed beneficial effects in the acute inflammatory phase.

Infection results in fibrinous or suppurative inflammatory damage along the epithelial surface of the uterine and the peritoneal surface of the fallopian tubes and ovaries, which leads to scarring, adhesions, and possibly partial or total obstruction of the uterine (Brotman, 2011). Previous studies have reported that uterine tissue adhesions can be observed pathologically after only 14 days of pelvic infection (Fan et al., 2021). However, treatment with levofloxacin or KFXYS for 14 days had a certain effect on reducing the adhesion area, the combination treatment was the most effective in protecting the uterine mucous membrane from inflammatory adhesion and reducing the adhesion area in the uterine mucosa.

Another potential health benefit of KFXYS treatment is provided its ability to protect the ultrastructure of the cell. The TEM results showed that PID caused a decrease in the number of microvilli on the epithelial cells and disappearance of the calyx. Microvilli are finger-like membrane protrusions supported by the actin cytoskeleton that are found on almost all cell types. A growing body of evidence suggests that microvilli, which have curved glycocalyces, play important roles in signal transduction leading to immune responses (Orbach and Su, 2020). KFXYS restored the microvilli on epithelial cells in the uterine mucosa and better protected the ultrastructural integrity of the cell to control inflammation. Mitochondria are influenced by a multitude of vital signals involved in the regulation of energy metabolism and cytosolic protein homeostasis, which prevent cell survival by modulating mitochondrial function and structure (Liu et al., 2018; Sedlackova and Korolchuk, 2019). In our experiment, we observed mitochondrial swelling and even rupture in the endometrial epithelium of rats with PID; however, after drug treatment, especially the combination therapy, mitochondrial swelling was significantly relieved, and the recovery of cell morphology was promoted and fat droplets were eliminated.

The transcription factor NF-κB is associated with a number of inflammatory signals, and the genes regulated by NF-κB play important roles in maintaining many aspects of cellular activity, such as immune responses to viral/bacterial infection, cell proliferation-survival-apoptosis, stress, and injury (Brockman et al., 1995; Sun, 2017). Generally, the inhibitory protein IκB interacts and binds with the p65 and p50 subunits of NF-κB through ankyrin repeat domains. In response to appropriate stimuli, such as TLR, IL-1β, or TNF-α, IκB kinase is activated and phosphorylates the p65 subunit (Lawrence, 2009). In our study, we observed that the ratio of p-p65/p65 was significantly increased in rats with PID at D14, indicating that NF-κB was activated during the acute PID stage. But the treatment with levofloxacin or KFXYS effectively reduced this activation.

With the progression of pelvic inflammation, endometrial fibrosis, which is the main pathological feature of intrauterine adhesion, begins to occur. Previous data from the literature suggest that endometrial fibrosis involves the accumulation of extracellular matrix (ECM) and that TGF-β/MMPs are central profibrotic mediators (Walton et al., 2017). As a regulator of the ECM, TGF-β plays a critical role in the development of fibrogenesis and organ dysfunction in a number of diseases, such as renal diseases and pulmonary fibrosis (Chegini, 2008; Hayden and Ghosh, 2004). Experimental and clinical data have shown that TGF-β is associated with the postsurgical intrauterine adhesion (Abudukeyoumu et al., 2020). In our study, we found a significant increase in the TGF-β level in uterine adhesions in PID model rats, which is consistent with previous reports showing that TGF-β is highly expressed in fibrotic tissues. And TGF-β expression was significantly reduced after treated with KFXYS or levofloxacin, especially after the combination therapy, indicating the efficacy of KFXYS in attenuating adhesion.

A large number of studies have shown that MMPs are key enzymes involved in ECM degradation and are strongly associated with fibrosis. MMP-2, also called gelatinase A (type IV collagenase; metallogelatinase), mainly acts on substrates including elastin, fibronectin and collagen type I and plays an important role in the process of collagen deposition in fibrotic diseases (Gaide Chevronnay et al., 2012). Increased MMP-2 expression is associated with fibrosis due to recurrent lung injury and inflammation (Arpino et al., 2015). In our experiment, the expression of MMP-2 was significantly down-regulated in adhesive uterine tissue in rats with PID, leading to accelerated accumulation of ECM and severe endometrial adhesion.

Taken together, this study first showed that KFXYS has a significant anti-inflammatory effect by inhibiting activation of the NF-κB pathway, and correspondingly reduced TGF-β/MMP-2 associated tissue adhesion, which suggests that KFXYS may be a potential adjunct therapeutic agent for PID, especially in patients with antibiotic resistance.

## Data Availability

The original contributions presented in the study are included in the article/Supplementary Material, further inquiries can be directed to the corresponding author.
