# Supplementary figures and images for: Kangfuxiaoyanshuan alleviates uterine inflammation and adhesion via inhibiting NF-κB p65 and TGF-β/MMP-2 signaling pathway in pelvic inflammatory disease rats
Source: Front Pharmacol. 2022 Jul 18;13:894149. doi: 10.3389/fphar.2022.894149 (PMC9340273; doi:10.3389/fphar.2022.894149)

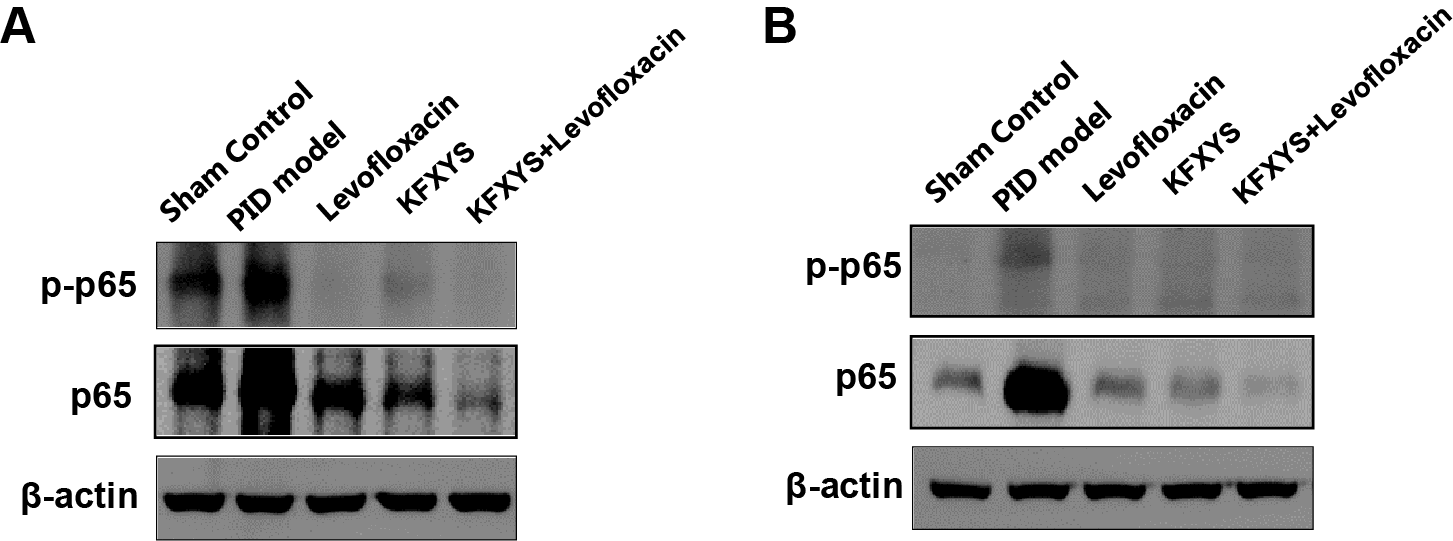

Supplement: Supplementary file 1 [file Image1.TIF]
